# Characterization of content associated with lesbian, gay, bisexual, transgender, intersex, and queer individuals in Chilean medical schools: a cross-sectional survey

**DOI:** 10.1186/s12909-024-05150-6

**Published:** 2024-02-21

**Authors:** Marcos Rojas, Joaquín Cánepa González, Nicolás Ortiz-López

**Affiliations:** 1 School of Education, Stanford University, California, United States of America; 2 School of Medicine, Faculty of Medicine, Universidad de Chile, Santiago, Chile; 3 Faculty of Medicine, Universidad de Chile, Av. Independencia 1027, Independencia, Santiago, Chile

**Keywords:** Curriculum, Gender minority, LGBTIQ, Medical education, Medical school, Undergraduate medical education

## Abstract

**Background:**

Lesbian, gay, bisexual, transgender, intersex, queer, and other sexual and gender identities (LGBTIQ+) individuals face health inequities. Additionally, medical students report a lack of confidence in providing specific health care to LGBTIQ + individuals, and medical schools do not offer the breadth and depth of coverage needed to fully prepare and make them comfortable in caring for these individuals. This study aims to characterize the teaching of curricular content related to LGBTIQ + health issues in medical schools in Chile.

**Methods:**

This was a cross-sectional descriptive mixed-methods study based on a 15-question survey sent to school directors of the 24 medical schools in Chile, conducted between October 2020 and July 2021. The questions included in the study were mostly based on two pre-existing questionnaires covering content, assessment methods, and identification of barriers to teaching this content.

**Results:**

The validated questionnaire was answered by 14 of 24 Chilean medical schools, with 11 schools (78.9%) declaring that they included some training in their curriculum. The predominant range of time allocated to LGBTIQ + training in medical programs was between 1 and 5 h. The most addressed topics were HIV (92.85%), sexual orientation (78.57%), and chronic disease risk in LGBTIQ + populations (78.57%). Most schools, accounting for 71.5%, considered the content they delivered to be “moderately insufficient” or “insufficient”. Regarding the teaching methodologies, the most used were lectures (92.8%), clinical cases (42.9%), and clinical simulation (28.6%).

**Conclusion:**

Most surveyed medical schools reported curricular spaces dedicated to teaching health issues of LGBTIQ + individuals, primarily during the pre-internship training period. However, the time allocated is insufficient, and there is little approach to topics beyond the patient’s sexual history or sexual orientation. Given the crucial role of medical schools, they must adopt both local and national strategies to enrich training focused on the care of LGBTIQ + patients.

**Supplementary Information:**

The online version contains supplementary material available at 10.1186/s12909-024-05150-6.

## Background

Lesbian, gay, bisexual, transgender, intersex, queer, and other sexual and gender identities (LGBTIQ+) individuals face health inequities. One significant aspect of these inequities is worse health outcomes, such as the increased risk of chronic diseases (e.g., asthma, diabetes, heart disease) [1], mental health conditions (e.g., eating disorders, mental illnesses, depression, and suicidal ideation) [1–3], substance abuse (alcohol, tobacco, and other drugs) [2, 4], different types of cancer (e.g., anal cancer, testicular cancer, breast cancer, cervical cancer) [5], and sexually transmitted infections (e.g., human immunodeficiency virus, gonorrhea, chlamydia, viral hepatitis) [6]. On the other hand, they face barriers to accessing the health system, such as heteronormative attitudes imposed by health professionals, homophobia, and discrimination, which generate less health system use by the LGBTIQ + populations [7].

In addition, medical students report a lack of confidence in providing specific health care to the LGBTIQ + individuals [8], and medical schools do not offer the breadth and depth of coverage they need to be fully prepared and comfortable to care for LGBTIQ + patients [9].

Recognizing this, many medical organizations have called for better training of health professionals to care for people with diverse sexual orientations, gender identities, and gender expressions [10, 11]. Medical education about LGBTIQ + individuals’ health is essential for the quality of health of these groups. It has been shown that medical students who received instruction on the sexuality and health of LGBTIQ + populations felt more prepared and comfortable when treating patients who identified as LGBTIQ + and better understood the clinical relevance of sexuality, orientation, and gender identity [12].

Internationally, studies show a narrow focus on LGBTIQ + content in medical schools across the United States, United Kingdom, Canada, Australia, South Africa, and Japan [8, 13–17]. Factors such as limited instructional time, discomfort with the topic, perceived relevance, and teacher expertise contribute to this issue [18].

There are currently no studies in Chile on content coverage of specific health issues of the LGBTIQ + populations.

## Methods

### Aim

This study aims to characterize the teaching of curricular content related to health issues of the LGBTIQ + populations in medical schools in Chile.

### Study design

This was a cross-sectional study employing a descriptive qualitative-quantitative, remote survey. We followed the recommendations of the Strengthening the Reporting of Observational Studies in Epidemiology (STROBE) protocol for reporting cross-sectional studies [19].

### Questionnaire design

We identified the items included in the survey from the literature and adapted them from two existing questionnaires [13, 15]. We combined the two questionnaires into a single version consisting of 14 questions, which we then translated into Spanish and adapted for the Chilean context. Content validation was performed by nine experts (three PhDs in Education, three Masters in Medical Education, two Sociologists, and one individual with a diploma in Gender and Diversity). Eight were university faculty in Chile, and one was from an NGO focused on promoting sexual and gender diversity inclusion in Chilean society. We evaluated the questionnaire by assessing each item using the expert judgment template proposed by Escobar-Pérez and Cuervo-Martínez [20], with ratings across four levels: does not meet the criteria, low level, moderate, and high level, based on the definition of indicators in correspondence with the characteristics to be evaluated: sufficiency, clarity, coherence, and relevance. Additionally, the expert’s qualitative observations led us to include two questions: Question 12 (What teaching methodology does your program use to achieve learning outcomes on LGBTIQ + topics for your students?) and Subquestion 8.12 (Non-binary gender identities (queer)) [21]. Finally, we conducted a pilot with nine professors to evaluate comprehensibility, acceptability, and application time.

### Data collection

In Chile, the medical program corresponds to an undergraduate study program with a duration of seven years and is overseen by a school director. The first five years, known as the pre-internship (or undergraduate) stage, involve pre-clinical and clinical courses, while the final two years constitute the internship stage. Upon completing Chile’s seven-year medical program, graduates obtain the degree equivalent to the Doctor of Medicine (M.D.).

In this study, we distributed the questionnaires to the directors of 24 medical schools in the country via email, using the addresses listed on the medical schools’ websites, and received the responses through the same means. If a medical school considered that another representative besides the school director would be more suitable to answer the survey, that individual was invited to participate. It is important to note that all respondents were professionals typically over 22 years old, reflecting the minimum age for holding a professional degree in medical education. We collected the study data between October 2020 and July 2021. Before answering the questionnaire, we obtained informed consent, requesting its signature alongside the email invitation. We requested each institution to answer only one questionnaire.

### Qualitative analysis

We analyzed free-text responses using a grounded theory-derived approach with no a priori defined assumptions [22]. We developed unique codes, indicative of common themes, for each question. All authors independently coded the free-text responses, with each response potentially receiving multiple codes. The authors then compiled their coding lists, requiring at least two authors’ agreement for inclusion in a specific code group.

### Ethics approval and consent to participate

We carried out all methods in accordance with the Declaration of Helsinki. The Research Ethics Committee of the University of Chile, School of Medicine, approved this study (Act No. 053, Project No. 063-2020). Participation in the study was voluntary and anonymous. We obtained written informed consent from each participant immediately before they completed the questionnaire. Additionally, we did not collect information on the names of the medical schools or any other details that could identify the universities.

## Results

### Questionnaire validation

We obtained a questionnaire containing 15 questions. Five of the 15 questions permitted free text input in addition to multiple-choice selections. The questionnaire is included in English and Spanish in Supplementary Appendix SA1. Nine experts validated the questionnaire confirming its sufficiency, clarity, coherence, and relevance (Supplementary Table S1). We made minor modifications for clarity after piloting it with nine university professors.

### Dedicated hours and coverage of curricular content associated with LGBTIQ + individuals

The response rate was 58.3% (14 of 24 medical schools). Among them, 11 schools (78.6%) declared that their program includes some degree of training regarding the health of the LGBTIQ + populations.

**Fig. 1:**
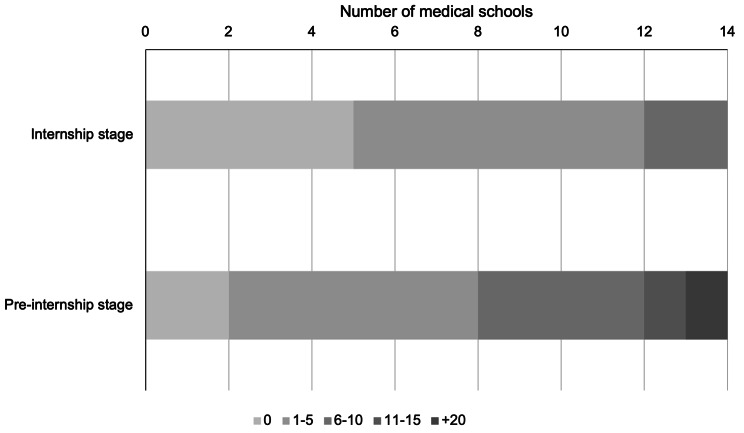
Hours Dedicated to Teaching LGBTIQ + Health Content in Chilean Medical Schools. Ranges express time from 0 to + 20 h

Regarding the number of hours invested in LGBTIQ + training at the pre-internship stage, the most common range reported by medical schools was ‘1–5 hours’. Notably, two medical schools indicated they allocated no time for this training, while one reported dedicating over 20 h. Similarly, during the internship stage, the ‘1–5 hours’ range remained the most prevalent, with five medical schools reporting no hours dedicated to LGBTIQ + training (Fig. 1). The distribution of these topics during medical training was asymmetric; 10 universities (71.4%) stated that these topics were at the pre-internship stage in different courses.

During the internship, eight universities (57.2%) reported that they carried out some teaching activities that included specific health content for LGBTIQ + individuals, while two medical schools offered spaces in an internship specifically designed to facilitate the care of LGBTIQ + patients. In 11 of the schools (78.6%), the curriculum included instruction on how to obtain information about relationships with people of the same sex, and in eight schools (57.1%), it encompassed teaching the difference between behavior and identity.

**Fig. 2:**
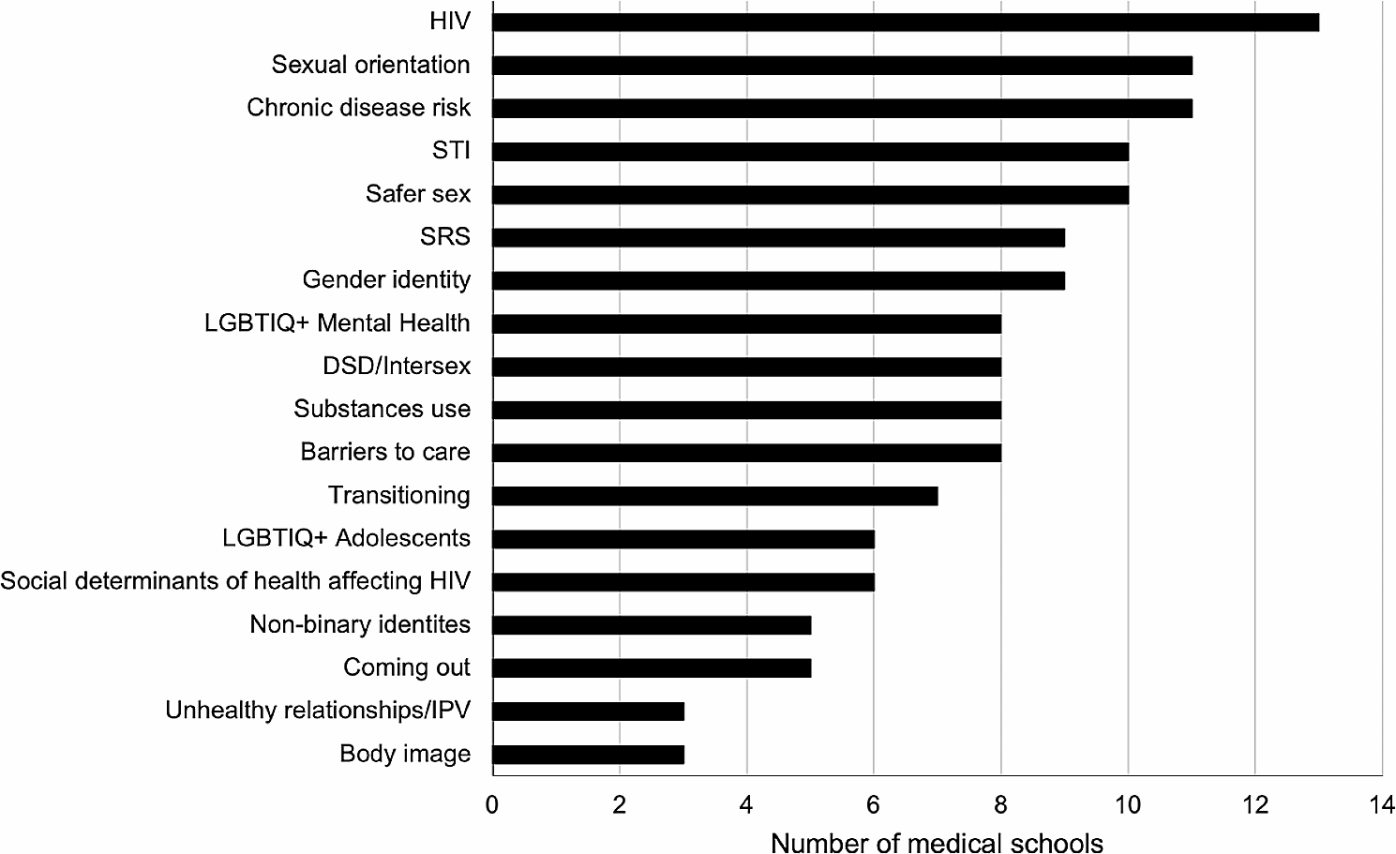
Coverage of LGBTIQ + Topics Taught During the Required Curriculum in Chilean Medical Schools. Abbreviations: DSD: disorders of sex development; HIV: human immunodeficiency virus; IPV: intimate partner violence; LGBTQ+: Lesbian, gay, bisexual, transgender, and queer; SRS: sex-reassignment surgery; STI: sexually transmitted infections

The medical schools indicated the coverage of content associated with LGBTIQ + individuals offered in the mandatory curriculum of their schools within a list of 18 topics (Fig. 2). The most addressed topics were HIV in 13 schools (92.85%), sexual orientation, and chronic disease risk in LGBTIQ + populations in 11 schools (78.57%); while the least addressed topics included non-binary identities and coming out in only five schools (35.71%), and unhealthy relationships/IPV and body image in LGBTIQ + populations in three schools (21.42%).

When openly asking about other topics that they would like to provide in their medical schools, two new categories emerged. The first category, related to LGBTIQ + health in different stages of the life cycle, was exemplified by Medical school 5’s input: ‘Health in different stages of the LGBTQ + individual and family life cycle’. The second category focused on health policies for LGBTIQ + populations, as highlighted by Medical school 6: ‘health programs in the public system for the LGBTQ + population’. The participating schools mentioned each category twice.

Regarding the opinion on the sufficiency of the topics delivered, 71.5% of the medical schools considered that they were “moderately insufficient” or “insufficient,” while 14.2% believed that they were “moderately sufficient” or “sufficient.” Regarding barriers to addressing these topics, 42.9% indicated that there were no concrete ideas about what to do, 42.9% considered that the curriculum was too tight to offer a class, and 35.7% commented that there were no suitable instructors for handling them. On the other hand, the strategies proposed to increase this content consisted of curricular material focused on the health of LGBTIQ + people/health inequities (92.9%) and more time allocated in the curriculum (57.1%).

### Teaching and assessment methods used

**Fig. 3:**
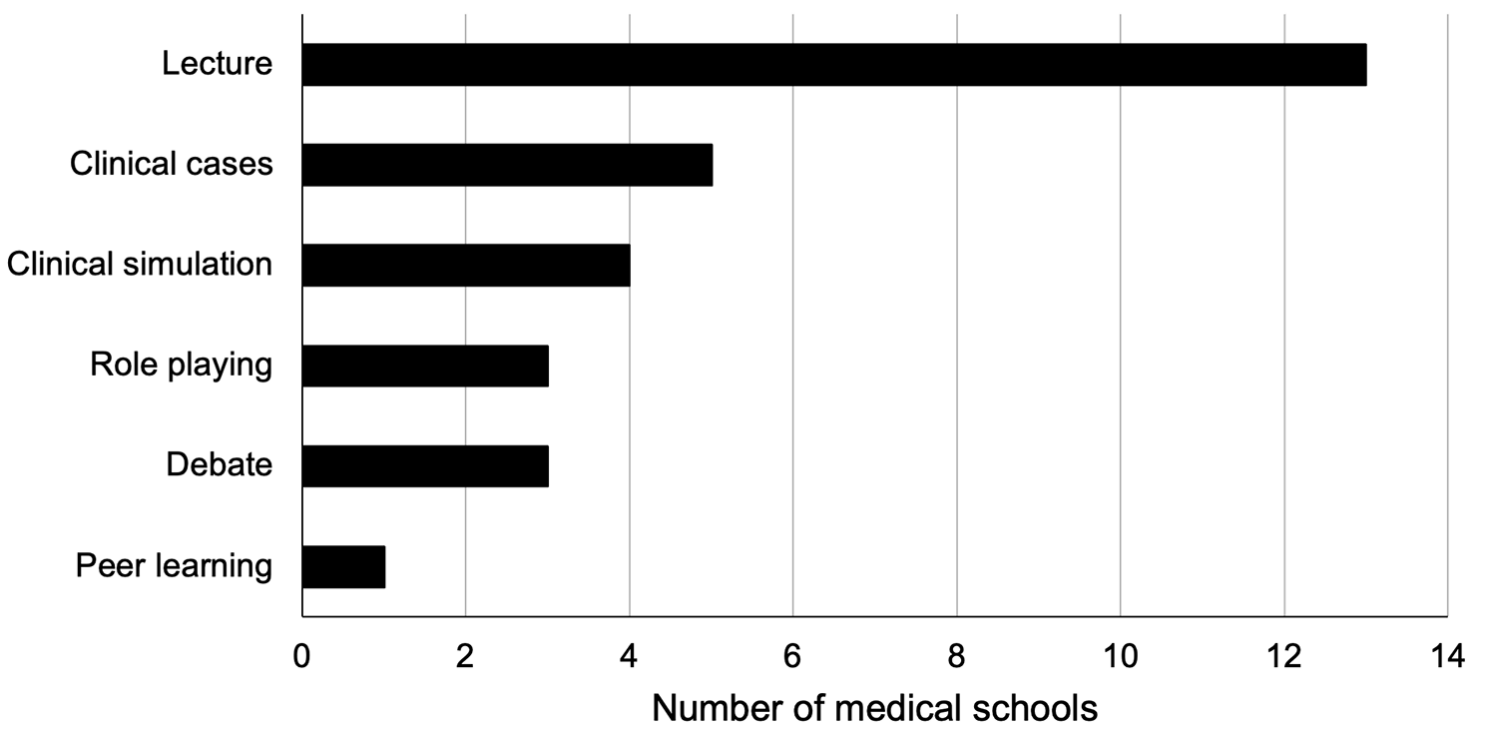
Teaching Methods for LGBTIQ + Topics in Chilean Medical Schools

**Fig. 4:**
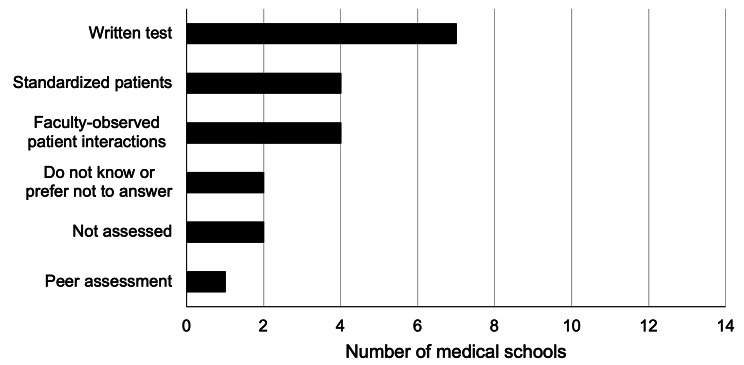
Assessment Methods for LGBTIQ + Topics in Chilean Medical Schools

The teaching methodologies for the achievement of knowledge of LGBTIQ + topics mainly included lectures in 13 schools (92.8%), clinical cases in five schools (42.9%), and clinical simulation in four schools (28.6%) (Fig. 3). Seven schools (50%) predominantly evaluated the topics through written tests, followed by four schools (28.5%) using standardized patients and faculty-observed patient interactions. Two schools did not evaluate these topics, and two did not know or preferred not to answer (Fig. 4).

## Discussion

This study aimed to characterize how medical schools in Chile integrate curricular content on LGBTIQ + health issues into their programs. Our focus encompassed both the pre-internship (first five years) and internship stages (final two years), which collectively form the mandatory training pathway for obtaining the M.D. degree in Chile. Unlike in many countries, Chilean medical schools oversee content in both stages, providing a unique perspective on the complete medical curriculum.

### Presence of LGBTIQ + content and hours of dedication

We found that 78.6% of medical schools reported teaching some related content. This percentage is above what was previously reported by other studies; for instance, only 27.5% of Japanese medical schools reported teaching some of these topics [15], which could be explained by contextual factors such as strong cultural taboos around sexuality and intimacy in Asia [23].

In Chile, the range of time allocated to LGBTIQ + training in medical programs predominantly fell between 1 and 5 h, both in pre-internship and internship stages. Specifically, during pre-internship, two medical schools reported no hours allocated for this training, and one school indicated over 20 h, while at the internship stage, five medical schools reported no hours dedicated to this training. This contrasts with findings from similar studies elsewhere: in the US and Canada a median of five hours was reported [13], while a study in the UK reported a median of 11 h [17]. Despite Chilean medical schools dedicating time to LGBTIQ + issues, the hours reported did not reach the minimum of 35 h considered necessary to achieve LGBTIQ + cultural competence in medical students, a standard that also includes interacting with at least 35 LGBTIQ + patients during their training [24].

### LGBTIQ + content present in medical training

The topics mainly corresponded to HIV from a biomedical perspective, other sexually transmitted infections (not HIV), and sexual orientation, while other topics such as non-binary identities, partner violence, or body image in the LGBTIQ + populations were among the least taught topics. These data highlight the need for a shift towards a more holistic approach to LGBTIQ + health medical education, moving beyond a strictly biomedical focus to include social determinants of health, as underscored in Cooper, Chacko, and Christner’s study on integrating LGBT health into medical curricula through the lens of social determinants [25].

The topics related to the trans population were among the least addressed by Chilean medical schools, including sex-reassignment surgery, gender identity, and transitioning. The deficit in this type of content has been previously reported. For example, a quarter of the students reported not feeling prepared to discuss gender reassignment surgery or gender transitioning, while 80% felt prepared to discuss HIV with their patients, according to a survey of 4,262 medical students from 170 schools in the United States and Canada [9]. Likewise, medical students have reported feeling less competent and comfortable treating transgender patients than lesbian, gay, and bisexual patients [26, 27]. Moreover, as medical students in the UK advance in their training, they become more confident in discussing with patients their sexual orientation, but not their gender identity [28].

Transgender patients experience discrimination from health professionals, decreasing their use of and access to these services [29]. Specifically, they reported being called by wrong names, being consulted about inappropriate topics, having their concerns dismissed, being insulted, ridiculed, being denied health care, and even feeling that they needed to educate the health professionals who cared for them [30, 31]. In addition, they experienced worse outcomes in their health care compared to their cisgender peers with higher rates of cancer, higher cardiovascular risk, higher presence of chronic diseases, sexually transmitted infections, substance use, and mental health conditions [31, 32]. Medical schools should make specific efforts around health for the transgender population. Biases present in basic science training, the production of biomedical knowledge, and the understanding of the categories of sex and gender as uncomplicated topics have been detected [33].

LGBTIQ + health at different life cycle stages emerged as an issue when asking about content not present in the survey. In the US, it was estimated that by 2030 the LGBT populations over 65 years of age could even double its number, reaching 6 million [34, 35]. LGB patients have a higher risk of disability, poorer mental health, a higher prevalence of smoking, and higher alcohol consumption than their heterosexual peers [36]. Elderly lesbian patients are less likely to access preventive screening with mammography, and trans patients, as they age, are more likely to have health problems related to their biological sex, which provides a stressor by having diseases of a sex with which they do not identify [37]. Therefore, medical schools must integrate LGBTIQ + content into their courses and internships dealing with geriatric patients.

### Curricular modifications

In this study, 71.5% of the medical schools believed that the content delivered regarding LGBTIQ + was moderately insufficient or insufficient. Identifying knowledge gaps regarding LGBTIQ + health content in undergraduate medical training is relevant since it has been shown that curricular modifications effectively increase medical students’ knowledge of LGBT health issues [38]. On the one hand, integrating the LGBTQ curriculum and patient exposure may reduce the risk of adverse health outcomes within this community [39]. On the other hand, literature has reported that medical students perceived insufficient curricular coverage of LGBTIQ + health issues, heterosexist assumptions in the curriculum, and even transphobic content in classes [40]. The curricular level’s limitations included the lack of an integrated curriculum and competent academics regarding these topics [28, 41].

In improving teaching content, it is important to incorporate the recommendations of LGBTIQ + community members into guidelines for working with LGBTIQ + patients [42]. Although curricular inclusions and modifications at the undergraduate level are essential, it is also necessary for medical schools to provide continuing medical education opportunities to current physicians to ensure that they all have the basic knowledge and skills since a lack of knowledge and competence has been demonstrated in these topics [43].

### Extracurricular modifications and the hidden curriculum

This study detected the presence of LGBTIQ + content in Chilean medical schools; however, it is essential to note that extracurricular measures related to the hidden curriculum impact doctors’ competence in these issues. What students acquire outside the classroom will be transferred to their formal education, and therefore it is relevant that the presence of these issues transcends university life [44].

The guidelines of the Association of American Medical Colleges for implementing curricular and institutional changes to improve the health of the LGBTIQ + populations indicate that it is crucial to establish environments in which reflection is promoted, research is carried out, and the complexity regarding gender and sexuality is understood [45]. The efforts made at the institutional culture level and in promoting respect and diversity have shown improved health students’ attitudes towards LGBTIQ + patients [46]. Additionally, clinical educators must understand the impact they make through their role models and seek to deliver training and care without prejudice [47, 48]. Finally, medical school administrators and faculty can contribute by creating an identity-affirming environment that not only helps LGBTIQ + students feel more included, but also extends this inclusivity to LGBTIQ + faculty and university employees, thereby reducing implicit biases among all members of the academic community, including those not part of sexual minorities [49–51].

### Methodologies of teaching and learning

The primary methodologies used by Chilean medical schools corresponded to lectures (92.8%), clinical cases (42.9%), and clinical simulation (28.6%). These findings align with what Obedin-Maliver et al. reported, where the principal methodology used in medical schools in the United States and Canada was lecturing (59.8%) [13]. Numerous methods are reported in the literature, including presentations, interview sessions, group work, panel discussions with LGBTIQ + populations, peer-to-peer learning, forums, and the use of virtual material [43].

Exposing students to LGBTIQ + patients can increase their comfort level in caring for these individuals [24], and Solotke et al. have recommended including LGBTIQ + topics in medical schools [52]. Similarly, medical students believe it would be beneficial to address LGBTIQ + issues in communication training sessions and through activities that directly involve LGBT patients [28]. Several studies have integrated direct interactions between medical students and LGBTIQ + patients, from panel discussions in pre-clinical courses [53, 54] to clinical clerkships at LGBT health centers [55]. However, Mains-Mason et al.‘s systematic review, which concentrated on studies assessing knowledge retention and/or clinical skills acquisition in medical trainees, revealed a significant gap in clinical skills development, particularly in real patient interactions. Despite improvements in knowledge retention, their review found that most curricula primarily utilized lectures or online modules, with minimal emphasis on interactive scenarios for enhancing communication skills with LGBTIQ + patients. This highlights the critical need for more practical, patient-centric experiences in medical education to effectively bolster both theoretical knowledge and clinical skill sets [56].

Regarding simulated patients, in the United States and Canada, there is an increase in the use of this methodology to represent LGBTIQ + patients and increase the exposure of students. However, there is no consensus on who should perform sexual minority patients [57].

### Strengths and limitations

A major strength of this study is that it is the first at the Latin American level to characterize the teaching of curricular content associated with the LGBTIQ + populations. In addition, it provides a validated instrument for measuring LGBTIQ + health topics and the current level of coverage in the context of Chilean medical schools. On the other hand, the survey was answered by the medical school directors or their delegates, who had the most detailed knowledge regarding the curriculum of the medical program at their school.

Within the limitations, for ethical reasons (i.e., guaranteeing anonymity in the participation in the context of a low number of participants), it was not possible to include segmentation variables about the participating medical schools; therefore, characterizing responders and non-responders was not feasible. Moreover, the subjects might have felt pressured to give socially desirable answers despite being an anonymous questionnaire. Finally, the response rate was 58.3%, so the sample might not represent all Chilean medical schools.

### Recommendations

This study revealed that Chilean medical schools used limited hours in teaching LGBTIQ + health content, especially in the internship stage of training. In addition, the topics taught were often restricted. Based on our findings and discussion, we suggest the following recommendations:

**Table 1:** Recommendations to improve LGBTIQ + health education in medical schools

Level of Implementation	Recommendations
National	National regulatory institutions should promote and monitor teaching LGBTIQ + topics in medical schools.
Institutional	Medical schools should locally assess the current knowledge of medical students on LGBTIQ + health topics to identify potential gaps. Schools should involve LGBTIQ + health experts in teaching activities and curricular decision-making. It’s crucial to ensure institutional coherence in both the treatment and care of the LGBTIQ + individuals within the educational community and what is effectively taught in the curriculum.
Curricular	Curricula should be enhanced with content addressing deficient areas in LGBTIQ + health, including trans and non-binary identities, topics related to intersex health, adolescence, and LGBTIQ + mental health. Medical schools should aim to integrate LGBTIQ + health content throughout the curriculum, rather than confining it to isolated classes. The focus on LGBTIQ + individuals health should shift from a purely biomedical perspective to a more comprehensive approach that includes social determinants of health. The teaching of LGBTIQ + content in the internship stage (also known as the clinical stage in some non-Chilean contexts) should be reinforced through supervised real or simulated patient interactions.

## Conclusions

Most medical schools reported having curricular spaces intended for teaching on health issues of LGBTIQ + individuals, mainly during the pre-internship stage. However, the time allocated is insufficient, and there is little emphasis on topics beyond the patient’s sexual history or sexual orientation. Given the crucial role that medical schools play in population health, they must take measures to improve training on LGBTIQ + health to provide appropriate care to the LGBTIQ + populations and ameliorate the health inequities they experience.

It is necessary to expand this research to other health programs since it will ensure that all professionals in this area are competent in these issues, which is the only way to achieve equity in health for all.

### Electronic supplementary material

Below is the link to the electronic supplementary material.

Supplementary Material 1

## Data Availability

The datasets generated and/or analyzed during the current study are not publicly available due to the conditions stated by the Ethics Committee to protect the identity of the participant institutions but are available from the corresponding author on reasonable request after being anonymized.
