# Correction: Characterization of content associated with lesbian, gay, bisexual, transgender, intersex, and queer individuals in Chilean medical schools: a cross-sectional survey

**DOI:** 10.1186/s12909-024-05248-x

**Published:** 2024-03-13

**Authors:** Marcos Rojas, Joaquín Cánepa González, Nicolás Ortiz-López

**Affiliations:** 1 School of Education, Stanford University, California, United States of America; 2 School of Medicine, Faculty of Medicine, Universidad de Chile, Santiago, Chile; 3 Faculty of Medicine, Universidad de Chile, Av. Independencia 1027, Independencia, Santiago, Chile

**Correction: BMC Medical Education (2024) 24:167**

10.1186/s12909-024-05150-6

Following publication of the original article [1], the authors informed us that Fig. 4 is missing, and instead of having the correct figure in that position, Fig. 3 is duplicated.

The original article has been corrected.
