# Supplementary material for: Characterization of content associated with lesbian, gay, bisexual, transgender, intersex, and queer individuals in Chilean medical schools: a cross-sectional survey
Source: BMC Med Educ. 2024 Feb 21;24:167. doi: 10.1186/s12909-024-05150-6 (PMC10882924; doi:10.1186/s12909-024-05150-6)
Supplement: Supplementary file 1 — Supplementary Material 1 [file 12909_2024_5150_MOESM1_ESM.docx]

**Supplementary Appendix SA1**

**Questionnaire in Spanish**

**Pregunta 1**

¿Su carrera de medicina incluye formación sobre salud de personas LGBTIQ+?

o Sí

o No

o No sé

o Prefiero no responder

**Pregunta 2.1**

¿Cuántas horas totales están dedicadas para enseñar contenidos de salud de personas LGBTIQ+ durante las siguientes etapas de formación académica? (Si su carrera no registra los contenidos a enseñar por número de horas estime lo más precisamente, por favor). Responda considerando la ETAPA PRE-INTERNADO (o licenciatura).

o 0 hrs

o 1-5 hrs

o 6-10 hrs

o 11-15 hrs

o 16-20 hrs

o +20 hrs

**Pregunta 2.2**

¿Cuántas horas totales están dedicadas para enseñar contenidos de salud de personas LGBTIQ+ durante las siguientes etapas de formación académica? (Si su carrera no registra los contenidos a enseñar por número de horas estime lo más precisamente, por favor). Responda considerando la ETAPA INTERNADO.

o 0 hrs

o 1-5 hrs

o 6-10 hrs

o 11-15 hrs

o 16-20 hrs

o +20 hrs

**Pregunta 3**

Por favor complete la siguiente oración: En el plan curricular obligatorio de la etapa pre-internados (o licenciatura), los contenidos específicos de salud de personas LGBTIQ+ son:

o Insertos en diferentes asignaturas del plan curricular

o Insertos en una única asignatura dedicada a contenidos LGBTIQ+

o Insertos en diferentes asignaturas del plan curricular como también en una asignatura dedicada a contenidos LGBTIQ+

o No están insertos en ninguna asignatura

o No sé

o Prefiero no responder

**Pregunta 4**

¿Su carrera realiza alguna actividad docente que incluya contenidos específicos de salud de personas LGBTIQ+ en el plan curricular obligatorio de la etapa de internado?

o Sí, en el plan curricular obligatorio

o Sí, pero no en el plan curricular obligatorio (e.g., internado electivo)

o No

o No sé

o Prefiero no responder

**Pregunta 5**

¿Hay algún espacio en un internado clínico que esté específicamente diseñado para facilitar la atención de pacientes LGBTIQ+ (por ejemplo, rotaciones en centros enfocados en personas LGBTIQ+)?

o Sí, es requisito en un internado obligatorio

o Sí, está disponible como un internado electivo

o Sí, es requisito en un internado obligatorio y también está disponible como un internado electivo

o No

o No sé

o Prefiero no responder

**Pregunta 6**

Cuando los estudiantes están aprendiendo a tomar una historia clínica sexual, ¿se les enseña cómo obtener información sobre relaciones con personas del mismo sexo? por ejemplo, preguntando “¿tienes relaciones sexuales con hombres, mujeres o ambos?”

o Sí

o No

o No sé

o Prefiero no responder

**Pregunta 7**

Se les enseña a los estudiantes de su carrera la diferencia entre comportamiento e identidad (por ejemplo, un hombre que puede tener sexo con otro hombre e identificarse como heterosexual).

o Sí, en el plan curricular obligatorio

o No

o No sé

o Prefiero no responder

**Pregunta 8**

¿Su carrera provee educación para sus estudiantes en las siguientes áreas en algún punto del currículum? (Las definiciones de los términos marcados con un asterisco “*” aparecen debajo de esta pregunta).

| 8.1 Barreras del acceso a atención médica de las personas LGBTIQ+ | o Sí, requerida en el currículum  o Disponible como electivo y no requerida en el currículum  o No en el currículum  o No sé  o Prefiero no responder |
| --- | --- |
| 8.2 Uso de alcohol, tabaco y otras drogas entre la población LGBTIQ+ | o Sí, requerida en el currículum  o Disponible como electivo y no requerida en el currículum  o No en el currículum  o No sé  o Prefiero no responder |
| 8.3 Estrategias para el sexo seguro para las personas LGBTIQ+ | o Sí, requerida en el currículum  o Disponible como electivo y no requerida en el currículum  o No en el currículum  o No sé  o Prefiero no responder |
| 8.4 Infecciones de transmisión sexual (no VIH) con enfoque en el riesgo de personas LGBTIQ+ | o Sí, requerida en el currículum  o Disponible como electivo y no requerida en el currículum  o No en el currículum  o No sé  o Prefiero no responder |
| 8.5 VIH con enfoque biomédico | o Sí, requerida en el currículum  o Disponible como electivo y no requerida en el currículum  o No en el currículum  o No sé  o Prefiero no responder |
| 8.6 VIH con enfoque de determinantes sociales de la salud en las personas LGBTIQ+ | o Sí, requerida en el currículum  o Disponible como electivo y no requerida en el currículum  o No en el currículum  o No sé  o Prefiero no responder |
| 8.7 Riesgo de enfermedades crónicas en las personas LGBTIQ+ | o Sí, requerida en el currículum  o Disponible como electivo y no requerida en el currículum  o No en el currículum  o No sé  o Prefiero no responder |
| 8.8 Orientación sexual* | o Sí, requerida en el currículum  o Disponible como electivo y no requerida en el currículum  o No en el currículum  o No sé  o Prefiero no responder |
| 8.9 “Salir del closet”* | o Sí, requerida en el currículum  o Disponible como electivo y no requerida en el currículum  o No en el currículum  o No sé  o Prefiero no responder |
| 8.10 Identidad de género* | o Sí, requerida en el currículum  o Disponible como electivo y no requerida en el currículum  o No en el currículum  o No sé  o Prefiero no responder |
| 8.11 Trastornos del desarrollo sexual/intersexualidad* | o Sí, requerida en el currículum  o Disponible como electivo y no requerida en el currículum  o No en el currículum  o No sé  o Prefiero no responder |
| 8.12 Identidades de género no binarias (queer) | o Sí, requerida en el currículum  o Disponible como electivo y no requerida en el currículum  o No en el currículum  o No sé  o Prefiero no responder |
| 8.13 Transición* (e.g. hombre-a-mujer, mujer-a-hombre) | o Sí, requerida en el currículum  o Disponible como electivo y no requerida en el currículum  o No en el currículum  o No sé  o Prefiero no responder |
| 8.14 Cirugía de reasignación de sexo* | o Sí, requerida en el currículum  o Disponible como electivo y no requerida en el currículum  o No en el currículum  o No sé  o Prefiero no responder |
| 8.15 Salud de adolescentes LGBTIQ+ | o Sí, requerida en el currículum  o Disponible como electivo y no requerida en el currículum  o No en el currículum  o No sé  o Prefiero no responder |
| 8.16 Salud mental en personas LGBTIQ+ | o Sí, requerida en el currículum  o Disponible como electivo y no requerida en el currículum  o No en el currículum  o No sé  o Prefiero no responder |
| 8.17 Imagen corporal en personas LGBTIQ+ | o Sí, requerida en el currículum  o Disponible como electivo y no requerida en el currículum  o No en el currículum  o No sé  o Prefiero no responder |
| 8.18 Problemas más frecuentes en las relaciones de pareja entre personas LGBTIQ+ | o Sí, requerida en el currículum  o Disponible como electivo y no requerida en el currículum  o No en el currículum  o No sé  o Prefiero no responder |

***Definiciones:**

-Orientación sexual: La autoidentidad de un individuo de atracción física o emocional. “Heterosexual”, “bisexual” y “homosexual” son orientaciones sexuales.

-Salir del closet: Un proceso de revelar la propia orientación sexual o identidad de género a uno mismo u otros.

-Identidad de género: Sentimiento de una persona de profunda identificación psicológica como hombre, mujer, transgénero, sin género, u otro género, que puede o no corresponder con el cuerpo de la persona o sexo asignado al nacer.

-Intersexual: Término utilizado para una variedad de condiciones en las cuales una persona nace con una anatomía sexual que no se ajusta a las definiciones típicas de hombre o mujer. También es conocido como trastornos del desarrollo sexual.

-Transición: El proceso a través del cual una persona modifica sus características físicas y/o forma de expresión de género para ser consistente con la identidad de género. Este proceso, también referido como “afirmación de género” puede incluir terapia hormonal, cirugía de reasignación de género u otros componentes, es generalmente conducida bajo supervisión médica basado en un ser de estándares desarrollados por profesionales médicos.

-Cirugía de reasignación de sexo: La cirugía de alteración genital que a veces se realizan individuos transgéneros para cambiar físicamente sus cuerpos para corresponder a su identidad de género.

**Pregunta 9**

Los ítems de las preguntas anteriores pueden no abarcar una lista completa de los tópicos de salud LGBTIQ+. Otros tópicos pueden incluir atención de pacientes geriátricos LGBTIQ+, salud reproductiva en personas LGBTIQ+, y uso de personas LGBTIQ+ como sujetos de estudio. Por favor, describa en su opinión la cobertura de los contenidos LGBTIQ+, en general, en su carrera.

o Suficiente

o Moderadamente suficiente

o Ni suficiente ni insuficiente

o Moderadamente insuficiente

o Insuficiente

**Pregunta 10**

Por favor, mencione otros tópicos relacionados a personas LGBTIQ+ que su carrera provee.

**Pregunta 11**

Por favor, mencione otros tópicos relacionados a personas LGBTIQ+ que a su carrera de medicina le gustaría proveer.

**Pregunta 12**

¿Qué metodología de enseñanza usa su carrera para el logro de aprendizajes de temáticas LGBTIQ+ a sus estudiantes?

- Clase expositiva/Clase masiva/cátedra
- Aprendizaje entre pares
- Aprendizaje basado en equipos
- Role playing
- Método de casos
- Simulación clínica
- Debate en el aula
- Otros:____

**Pregunta 13**

¿Qué método usa su carrera para evaluar el logro de los aprendizajes de temáticas LGBTIQ+ a sus estudiantes cuando se enseña?

- Prueba escrita (independiente del tipo de preguntas utilizadas)
- Interacciones con pacientes supervisadas por un profesor
- Evaluación entre pares
- Evaluación con pacientes estandarizados (actores)
- Evaluación por pacientes
- No se evalúa
- Prefiero no responder
- Otros:____

**Pregunta 14**

¿Qué barreras considera usted que tiene su carrera para aumentar el abordaje de los contenidos de salud de personas LGBTIQ+?

- No existen ideas concretas sobre qué hacer
- El plan de estudios es demasiado ajustado para ofrecer una clase
- No hay instructores adecuados
- Incapacidad de encontrar materiales didácticos estándar
- La universidad no tiene una política sobre educación sexual y de minorías de género
- Actualmente no se ha identificado ninguna necesidad
- Otros:____

**Pregunta 15**

¿Cuáles estrategias piensa que son o podrían ser exitosas para aumentar los contenidos de salud de personas LGBTIQ+ en su carrera? (por favor, marque todas las que aplican)

- Material curricular enfocado en salud de personas LGBTIQ+/inequidades en salud
- Voluntad y capacidad de los profesores para enseñar contenidos LGBTIQ+
- Aumentar recursos financieros
- Apoyo logístico para enseñar contenidos LGBTIQ+
- Más tiempo en el currículum para poder enseñar contenidos LGBTIQ+
- Mayor investigación basada en la evidencia respecto a la salud de personas LGBTIQ+
- Cobertura curricular requerida por cuerpos acreditadores
- Preguntas basadas en salud LGBTIQ+/inequidades en salud en pruebas nacionales (e.g. EUNACOM)
- Métodos para evaluar el contenido curricular LGBTIQ+
- No sé
- Prefiero no responder
- Otros:____

**Questionnaire in English (The translation to English was conducted by the authors, and the questions need additional validation for adaptation to various contexts).**

**Question 1**

Does your medical program include training on LGBTIQ+ people's health?

o Yes

o No

o I don't know

o Prefer not to answer

**Question 2.1**

How many total hours are dedicated to teaching LGBTIQ+ health content during the following stages of academic training? (If your program does not record the content taught by the number of hours, please estimate as accurately as possible). Answer considering the PRE-INTERNSHIP STAGE (or undergraduate level).

o 0 h

o 1-5 h

o 6-10 h

o 11-15 h

o 16-20 h

o +20 h

**Question 2.2**

How many total hours are dedicated to teaching LGBTIQ+ health content during the following stages of academic training? (If your program does not record the content taught by the number of hours, please estimate as accurately as possible). Answer considering the INTERNSHIP STAGE.

o 0 h

o 1-5 h

o 6-10 h

o 11-15 h

o 16-20 h

o +20 h

**Question 3**

Please complete the following sentence: In the mandatory curricular plan of the pre-internship stage (or undergraduate level), the specific contents on LGBTIQ+ health are:

o Integrated into different courses of the curriculum

o Integrated into a single course dedicated to LGBTIQ+ content

o Integrated into different courses of the curriculum as well as in a course dedicated to LGBTIQ+ content

o Not integrated into any course

o I don't know

o Prefer not to answer

**Question 4**

Does your program conduct any teaching activity that includes specific LGBTIQ+ health content in the mandatory curricular plan during the internship stage?

o Yes, in the mandatory curricular plan

o Yes, but not in the mandatory curricular plan (e.g., elective internship)

o No

o I don't know

o Prefer not to answer

**Question 5**

Is there a specific space in a clinical internship designed to facilitate the care of LGBTIQ+ patients (for example, rotations in centers focused on LGBTIQ+ individuals)?

o Yes, it is required in a mandatory internship

o Yes, it is available as an elective internship

o Yes, it is required in a mandatory internship and also available as an elective internship

o No

o I don't know

o Prefer not to answer

**Question 6**

When students are learning to take a sexual history, are they taught how to obtain information about same-sex relationships? For example, by asking "Do you have sexual relations with men, women, or both?"

o Yes

o No

o I don't know

o Prefer not to answer

**Question 7**

Are students in your program taught the difference between behavior and identity (for example, a man who may have sex with another man but identifies as heterosexual)?

o Yes, in the mandatory curricular plan

o No

o I don't know

o Prefer not to answer

**Question 8**

Does your program provide education for its students in the following areas at some point in the curriculum? (Definitions of the terms marked with an asterisk "*" appear below this question).

| 8.1 Barriers to healthcare access for LGBTIQ+ individuals | o Yes, required in the curriculum  o Available as an elective and not required in the curriculum  o Not in the curriculum  o I don't know  o Prefer not to answer |
| --- | --- |
| 8.2 Alcohol, tobacco, and other drug use among the LGBTIQ+ population | o Yes, required in the curriculum  o Available as an elective and not required in the curriculum  o Not in the curriculum  o I don't know  o Prefer not to answer |
| 8.3 Strategies for safe sex for LGBTIQ+ individuals | o Yes, required in the curriculum  o Available as an elective and not required in the curriculum  o Not in the curriculum  o I don't know  o Prefer not to answer |
| 8.4 Sexually transmitted infections (non-HIV) focusing on the risk for LGBTIQ+ individuals | o Yes, required in the curriculum  o Available as an elective and not required in the curriculum  o Not in the curriculum  o I don't know  o Prefer not to answer |
| 8.5 HIV with a biomedical approach | o Yes, required in the curriculum  o Available as an elective and not required in the curriculum  o Not in the curriculum  o I don't know  o Prefer not to answer |
| 8.6 HIV with a focus on social determinants of health in LGBTIQ+ individuals | o Yes, required in the curriculum  o Available as an elective and not required in the curriculum  o Not in the curriculum  o I don't know  o Prefer not to answer |
| 8.7 Risk of chronic diseases in LGBTIQ+ individuals | o Yes, required in the curriculum  o Available as an elective and not required in the curriculum  o Not in the curriculum  o I don't know  o Prefer not to answer |
| 8.8 Sexual orientation* | o Yes, required in the curriculum  o Available as an elective and not required in the curriculum  o Not in the curriculum  o I don't know  o Prefer not to answer |
| 8.9 “Coming out”* | o Yes, required in the curriculum  o Available as an elective and not required in the curriculum  o Not in the curriculum  o I don't know  o Prefer not to answer |
| 8.10 Gender identity* | o Yes, required in the curriculum  o Available as an elective and not required in the curriculum  o Not in the curriculum  o I don't know  o Prefer not to answer |
| 8.11 Disorders of sexual development/intersexuality* | o Yes, required in the curriculum  o Available as an elective and not required in the curriculum  o Not in the curriculum  o I don't know  o Prefer not to answer |
| 8.12 Non-binary gender identities (queer) | o Yes, required in the curriculum  o Available as an elective and not required in the curriculum  o Not in the curriculum  o I don't know  o Prefer not to answer |
| 8.13 Transition* (e.g., man-to-woman, woman-to-man) | o Yes, required in the curriculum  o Available as an elective and not required in the curriculum  o Not in the curriculum  o I don't know  o Prefer not to answer |
| 8.14 Sex reassignment surgery* | o Yes, required in the curriculum  o Available as an elective and not required in the curriculum  o Not in the curriculum  o I don't know  o Prefer not to answer |
| 8.15 LGBTIQ+ adolescent health | o Yes, required in the curriculum  o Available as an elective and not required in the curriculum  o Not in the curriculum  o I don't know  o Prefer not to answer |
| 8.16 Mental health in LGBTIQ+ individuals | o Yes, required in the curriculum  o Available as an elective and not required in the curriculum  o Not in the curriculum  o I don't know  o Prefer not to answer |
| 8.17 Body image in LGBTIQ+ individuals | o Yes, required in the curriculum  o Available as an elective and not required in the curriculum  o Not in the curriculum  o I don't know  o Prefer not to answer |
| 8.18 Common problems in relationships among LGBTIQ+ individuals | o Yes, required in the curriculum  o Available as an elective and not required in the curriculum  o Not in the curriculum  o I don't know  o Prefer not to answer |

***Definitions:**

-Sexual Orientation: An individual's self-identity of physical or emotional attraction. "Heterosexual," "bisexual," and "homosexual" are sexual orientations.

-Coming Out: A process of revealing one's sexual orientation or gender identity to oneself or others.

-Gender Identity: A person's deep psychological identification as male, female, transgender, genderless, or another gender, which may or may not correspond with the person's body or sex assigned at birth.

-Intersex: A term used for a variety of conditions in which a person is born with sexual anatomy that does not fit typical definitions of male or female. Also known as disorders of sexual development.

-Transition: The process through which a person modifies their physical characteristics and/or gender expression to align with their gender identity. This process, also referred to as "gender affirmation," may include hormone therapy, gender reassignment surgery, or other components, and is generally conducted under medical supervision based on a set of standards developed by medical professionals.

-Sex Reassignment Surgery: Genital alteration surgery sometimes undergone by transgender individuals to physically change their bodies to match their gender identity.

**Question 9**

The items from the previous questions may not cover a complete list of LGBTIQ+ health topics. Other topics can include care for LGBTIQ+ geriatric patients, reproductive health in LGBTIQ+ individuals, and the use of LGBTIQ+ individuals as study subjects. Please describe, in your opinion, the overall coverage of LGBTIQ+ content in your program.

o Sufficient

o Moderately sufficient

o Neither sufficient nor insufficient

o Moderately insufficient

o Insufficient

**Question 10**

Please mention other topics related to LGBTIQ+ individuals that your program provides.

**Question 11**

Please mention other topics related to LGBTIQ+ individuals that your medical program would like to provide.

**Question 12**

What teaching methodology does your program use for achieving learning outcomes on LGBTIQ+ themes for your students?

- Expository class/lecture
- Peer learning
- Team-based learning
- Role playing
- Case method/Clinical cases
- Clinical simulation
- Debate
- Other:____

**Question 13**

What method does your program use to assess the achievement of learning outcomes on LGBTIQ+ themes for your students when taught?

- Written test (regardless of the type of questions used)
- Faculty-observed patient interactions
- Peer assessment
- Assessment with standardized patients (actors)
- Evaluation by patients
- Not assessed
- Prefer not to answer
- Other:____

**Question 14**

What barriers do you think your program faces in increasing the coverage of LGBTIQ+ health content?

- No concrete ideas on what to do
- Curriculum too tight to offer a class
- No suitable instructors
- Inability to find standard teaching materials
- The university does not have a policy on sexual and gender minority education
- No need identified currently
- Other:____

**Question 15**

What strategies do you think are or could be successful in increasing LGBTIQ+ health content in your program? (please mark all that apply)

- Curricular material focused on LGBTIQ+ health/health inequities
- Willingness and ability of teachers to teach LGBTIQ+ content
- Increased financial resources
- Logistical support for teaching LGBTIQ+ content
- More time in the curriculum to teach LGBTIQ+ content
- More evidence-based research on LGBTIQ+ health
- Curricular coverage required by accrediting bodies
- LGBTIQ+ health/health inequities based questions in national tests (e.g., EUNACOM)
- Methods for evaluating LGBTIQ+ curricular content
- I don't know
- Prefer not to answer
- Other:____

**Supplementary Table S1**

Validation results. The mean validation results are represented in the following table for each item in the questionnaire. 9 experts evaluated and validated the questionnaire, giving points according to each criteria of sufficiency, clarity, coherence and relevance. 1 point: criteria not met; 2 points: low level of accomplishment; 3 points: moderate level of accomplishment; 4 points: high level of accomplishment. The two expert-suggested questions, added after validation, are not included in this validation assessment.

|  | **Sufficiency** | **Coherence** | **Relevance** | **Clarity** |
| --- | --- | --- | --- | --- |
| **Item** | **Mean (SD)** | **Mean (SD)** | **Mean (SD)** | **Mean (SD)** |
| 1 | 3.33 (1.12) | 3.78 (0.44) | 4.00 (0.0) | 3.33 (1.00) |
| 2.1 | 3.44 (1.13) | 3.89 (0.33) | 3.67 (0.71) | 2.89 (0.78) |
| 2.2 | 3.44 (1.13) | 3.89 (0.33) | 3.33 (1.12) | 2.89 (1.05) |
| 3 | 3.67 (0.50) | 3.78 (0.44) | 4.00 (0.00) | 3.22 (0.83) |
| 4 | 3.44 (1.01) | 3.44 (1.01) | 3.67 (1.00) | 3.22 (1.09) |
| 5 | 3.78 (0.67) | 3.78 (0.67) | 3.89 (0.33) | 3.56 (0.73) |
| 6 | 3.56 (1.01) | 3.78 (0.44) | 4.00 (0.00) | 3.11 (1.17) |
| 7 | 3.89 (0.33) | 4.00 (0.00) | 4.00 (0.00) | 3.67 (0.50) |
| 8.1 | 4.00 (0.00) | 4.00 (0.00) | 4.00 (0.00) | 3.89 (0.33) |
| 8.2 | 4.00 (0.00) | 4.00 (0.00) | 4.00 (0.00) | 4.00 (0.00) |
| 8.3 | 4.00 (0.00) | 4.00 (0.00) | 4.00 (0.00) | 3.89 (0.33) |
| 8.4 | 4.00 (0.00) | 4.00 (0.00) | 4.00 (0.00) | 3.89 (0.33) |
| 8.5 | 4.00 (0.00) | 4.00 (0.00) | 3.56 (1.33) | 3.78 (0.67) |
| 8.6 | 4.00 (0.00) | 4.00 (0.00) | 4.00 (0.00) | 4.00 (0.00) |
| 8.7 | 3.78 (0.67) | 3.78 (0.67) | 3.78 (0.67) | 3.67 (1.00) |
| 8.8 | 4.00 (0.00) | 4.00 (0.00) | 4.00 (0.00) | 3.89 (0.33) |
| 8.9 | 4.00 (0.00) | 3.78 (0.67) | 3.67 (0.71) | 3.67 (0.71) |
| 8.10 | 4.00 (0.00) | 4.00 (0.00) | 4.00 (0.00) | 3.89 (0.33) |
| 8.11 | 4.00 (0.00) | 4.00 (0.00) | 4.00 (0.00) | 3.89 (0.33) |
| 8.12 | 4.00 (0.00) | 4.00 (0.00) | 4.00 (0.00) | 3.89 (0.33) |
| 8.13 | 4.00 (0.00) | 4.00 (0.00) | 4.00 (0.00) | 3.89 (0.33) |
| 8.14 | 4.00 (0.00) | 4.00 (0.00) | 4.00 (0.00) | 3.89 (0.33) |
| 8.15 | 4.00 (0.00) | 4.00 (0.00) | 4.00 (0.00) | 4.00 (0.00) |
| 8.16 | 4.00 (0.00) | 4.00 (0.00) | 4.00 (0.00) | 4.00 (0.00) |
| 8.17 | 4.00 (0.00) | 3.78 (0.67) | 4.00 (0.00) | 3.44 (1.01) |
| 9 | 3.78 (0.67) | 3.78 (0.67) | 3.67 (1.00) | 3.22 (1.30) |
| 10 | 4.00 (0.00) | 4.00 (0.00) | 4.00 (0.00) | 3.44 (1.01) |
| 11 | 3.44 (1.33) | 3.88 (0.35) | 3.56 (1.33) | 2.67 (1.58) |
| 12 | 3.56 (0.88) | 3.78 (0.67) | 3.33 (1.32) | 3.56 (0.73) |
| 13 | 4.00 (0.00) | 4.00 (0.00) | 4.00 (0.00) | 3.44 (0.53) |
| 14 | 4.00 (0.00) | 4.00 (0.00) | 4.00 (0.00) | 3.22 (1.09) |

SD: Standard deviation
